# Environmental and occupational respiratory diseases – 1055. CD23, TH1/TH2 cytokines in children with bronchial asthma, bronchiolitis and bronchial pneumonia

**DOI:** 10.1186/1939-4551-6-S1-P53

**Published:** 2013-04-23

**Authors:** Anchoju Vijayendra Chary

**Affiliations:** 1 Microbiology and Immunology, National Institute of Nutrition (ICMR), Hyderabad, India

## Background

CD23 (FcεRII), is the low-affinity receptor for IgE and considered as a multifunctional cytokine. Soluble CD23 (sCD23) plays an important role in IgE synthesis. The aim of this study was to determine sCD23 and Th1, Th2 cytokines levels in Children with asthma, bronchiolitis and bronchial pneumonia.

## Methods

CD23, Histamine release, total IgE and various Th1, Th2 cytokines were determined in blood samples of patients with bronchial asthma (n = 23), bronchiolitis (n = 20) and bronchial pneumonia (n =20) and age & sex matched normal children (n = 20) were taken as controls.

## Results

Serum sCD23 was significantly increased (p<0.01) in bronchial asthma (1209.8 ± 68.01 pg/mL), bronchiolitis (1455.52 ± 146.92 pg/mL) and bronchial pneumonia (1406.35 ± 98.26 pg/mL) when compared to controls (691.5 ± 74.94 pg/mL). Serum IgE and blood histamine levels were increased significantly (P<0.05) and IFN-γ (Th1 cytokine) was significantly lower (P<0.01) in both bronchial asthma and bronchiolitis than in controls where as IFN-γ (Th1 cytokine) increased significantly in bronchial pneumonia compared to the other three groups.

## Conclusions

Our observations provide evidence on CD23 expression in children with and without asthma and a preferential activation of Th2 (IL-5) and suppression of Th1 (IFN- γ) cytokine in children with asthma. Comparable CD23 response in children with bronchial pneumonia and bronchial asthma, suggests nonspecific nature of CD23.

